# Educating Men—Compulsory Reading in the Enlightenment of Gender Diversity

**DOI:** 10.3389/jaws.2022.11063

**Published:** 2022-12-08

**Authors:** Andrew de Beaux

**Affiliations:** ^1^ Spire Murrayfield Hospital, Edinburgh, United Kingdom; ^2^ The University of Edinburgh, Edinburgh, United Kingdom

**Keywords:** men, women, gender, diversity, inequality

I was raised in a loving home, with a dominant, powerful and at times opinionated mother, and two similar older sisters. My dad, my brother and I had no chance. Now married with two sons, we think the odds at home are about even. With this background, I failed to see the issues about gender inequality, and in failing to see them, I was inherently sexist, call it “antiwomen in surgery.” At times it came out, was overt, and I am sure it caused offence. For that I apologies unreservedly.

This editorial is written in honour of my mentors, some older, some younger than me, who all shall remain nameless. But I thank them for educating me, taking me on a journey which I hope many other of my male colleagues will also walk. Not to tick a box, but to believe in, and tackle diversity in all its forms. I would like to share with you, my learning material.

As a surgeon myself, I would like to think of my intellectual prowess. So it was with some chagrin, that the first two books on my syllabus, were largely picture books, akin to comics. The first, “The trouble with women,” was a defining text (1). It is described as “a brilliantly witty book of cartoons, it reveals some of our greatest thinkers’ baffling theories about women.” Examples are drawn from history of great women, often under recognised because they were women, their research not publishable as they were women, and their work “stolen” by men and published to great acclaim. The second in the picture book series, was “Fruit of knowledge” (2). It explores the cultures and traditions that have shaped women’s health and beyond. At times, almost a sex manual, but that would denigrate the powerful messages in the book. The author uses the comics medium to reveal some very uncomfortable truths about how far we haven’t come.

The final book in the introductory or basics educating men course, is “We should all be feminists” (3). I am sure all of you will have a stereotypical view of a “feminist.” I had too, but this book so cleverly and expertly in a few sentences changed my view. I leave you to the pleasure of it changing your view as well.

From this point, there is an explosion in books you could read. Many biographical works of great women, some famous, others not so. But they may not always help you change or refine your view on women as leaders, scientists, surgeons at the very top of their game, or indeed doing a sterling job in whatever their position. I would like to share with you 3 other titles to add to your essential reading list. Let’s call it the “intermediate syllabus;”

“Inferior. The true power of women and the science that shows it” (4) is an interesting treatise of the present-day research on sex/gender differences. It exposes the fascinating and at times absurdity around the research into male/female differences. For centuries this research seems to focus on a relatively small number of differences identified between males and females, rather than on the many similarities. This obsession with the differences has perhaps led to decisions that have not been good for this world in general or its people that we share the earth with. Yes, women may in general be smaller, and thus have “smaller brains,” but we so easily fall into the trap of quantity over quality.

“Invisible women” (5) has to be next. A remarkable essay on how women have been forgotten about in so many aspects of everyday life. City planning, safe car seat design, you name it, the design was based around male shapes, sizes and traditional male work patterns. So what you may say? But for example, women in a car, in any seat, are more likely to die in a road traffic accident than their male equivalent under identical accident conditions.

And my final recommendation, was perhaps the book that flipped my thinking patterns, into the power of women, and perhaps better, the power of men and women, let’s call them people, working together for the better, using their combined skills. “x+y, A mathematician’s manifesto for rethinking gender” (6). We talk of masculine characteristics in men as good, and feminine traits in men as not good, and *vice versa*. But there are times when ‘masculine traits’ in either a man or woman are useful for the task in hand. Degenderising such traits, does allow advertising and employing the right person for the job! With the logic and clarity of a mathematician, we get a different view of the world optimising the place for everyone based more on their individual real talents, than stereotypical opinions.

Diversity thankfully is here to stay. We fight it, as humans tend to do about any change. But I leave you with an image that also has changed my view of people. It was the picture of a small child, wearing a T-shirt that had written on it;

Birthplace: earth.
Race: human.
Politics: freedom.
Religion: love.

I hope I, and anyone who has kindly read this far, continues to read and explore the reasons why we are like we are today, and when change for the better is needed, become a soldier to the cause. I am NO feminist. But I believe in the need to redress injustice, and gender equity is good and right for this world.
